# Surveillance for Mosquitoborne Transmission of Zika Virus, New York City, NY, USA, 2016

**DOI:** 10.3201/eid2405.170764

**Published:** 2018-05

**Authors:** Amanda Wahnich, Sandhya Clark, Danielle Bloch, Hannah Kubinson, Gili Hrusa, Dakai Liu, Jennifer L. Rakeman, Bisram Deocharan, Lucretia Jones, Sally Slavinski, Alaina Stoute, Robert Mathes, Don Weiss, Erin E. Conners, Sentinel Surveillance Working Group

**Affiliations:** New York City Department of Health and Mental Hygiene, Queens, New York, USA

**Keywords:** Zika virus, autochthonous, United States, New York, arbovirus, viruses, surveillance

## Abstract

A large number of imported cases of Zika virus infection and the potential for transmission by *Aedes albopictus* mosquitoes prompted the New York City Department of Health and Mental Hygiene to conduct sentinel, enhanced passive, and syndromic surveillance for locally acquired mosquitoborne Zika virus infections in New York City, NY, USA, during June–October 2016. Suspected case-patients were those >5 years of age without a travel history or sexual exposure who had >3 compatible signs/symptoms (arthralgia, fever, conjunctivitis, or rash). We identified 15 suspected cases and tested urine samples for Zika virus by using real-time reverse transcription PCR; all results were negative. We identified 308 emergency department visits for Zika-like illness, 40,073 visits for fever, and 17 unique spatiotemporal clusters of visits for fever. We identified no evidence of local transmission. Our experience offers possible surveillance tools for jurisdictions concerned about local mosquitoborne Zika virus or other arboviral transmission.

## Abstract 2

Sentinel, enhanced passive, and syndromic surveillance in 2016 did not identify any evidence of transmission.

Zika virus, an arbovirus of the genus *Flavivirus*, has spread rapidly across Latin America and the Caribbean region after an epidemic was identified in Brazil in early 2015 (*1**,**2*). Although it is usually clinically mild or asymptomatic, Zika virus infection during pregnancy can cause microcephaly and other severe brain, eye, and ear defects in the fetus (*3**,**4*). Among adults, Zika virus has also been linked to neurologic disorders, including Guillain-Barré syndrome (*5*).

The primary vector of the Zika virus epidemic, the mosquito *Aedes aegypti*, has not been found in New York City (NYC), NY, USA; however, the less-efficient mosquito vector *Ae. albopictus* is present throughout the city (*6**–**11*). Historically, NYC has not had local transmission of either dengue or chikungunya viruses, which are also spread by *Ae. aegypti and Ae. albopictus* mosquitoes.

NYC is a destination for a large population of travelers, as well as being the home of ≈1.8 million persons from the Caribbean region and Latin America, who might travel back and forth to Zika-affected areas (*12**–**14*). As of June 17, 2016, there were 182 confirmed cases of Zika virus infection in NYC, one of the highest case burdens in the United States (*15*).

The Centers for Disease Control and Prevention (CDC) interim response plan for Zika recommends enhanced surveillance in areas with *Ae. aegypti* mosquitoes (*16*). The risk for local mosquitoborne transmission in NYC was thought to be less than in jurisdictions with *Ae. aegypti* mosquitoes. However, local transmission was still a concern given the high number of travel-associated cases, the nascent knowledge about Zika transmission, and the potential need to rapidly implement local control measures should mosquitoborne transmission be demonstrated (*17*). In response, the NYC Department of Health and Mental Hygiene (DOHMH) enhanced both human surveillance and mosquito control efforts during 2016 (*15*). This report describes the establishment and outcomes of sentinel, enhanced citywide passive, and emergency department (ED) syndromic surveillance systems to identify potential human cases of local mosquitoborne transmission of Zika virus.

## Methods

### Sentinel Surveillance

DOHMH identified the first travel-associated Zika cases in NYC in January 2016 (*15*); the number of cases peaked in June 2016 (NYC DOHMH, unpub. data). Sentinel surveillance for local transmission was initiated in June 2016. Sentinel surveillance relies on detection of disease in facilities likelier to see cases, can require fewer public health resources than population-based surveillance, and can provide more detailed data than passive reporting (*18*). We selected facilities throughout the city for the sentinel surveillance network on the basis of locations in neighborhoods with high counts of reported cases of travel-associated Zika virus infection, historically elevated counts of travel-associated dengue or chikungunya diseases from Zika-affected countries, an environmental habitat conducive to *Ae. albopictus* mosquito breeding, or areas with large adult *Ae. albopictus* mosquito populations.

Participating sentinel clinical sites received patient screening criteria, reporting instructions, sterile urine collection tubes, educational posters, and, in some cases, mosquito repellents to distribute to persons planning to travel to Zika-affected areas. Sentinel sites used a paper DOHMH reporting form to capture clinical and demographic information on suspected cases.

One designated healthcare staff member at each site received weekly DOHMH update emails and was responsible for disseminating the sentinel case definition and any relevant updates to other providers onsite. These providers were of varying medical specialties, including internal medicine, emergency medicine, infectious disease, family medicine, and pediatrics. For assistance, providers could also contact the regular DOHMH 24-hour on-call physician or a direct sentinel call number active during the surveillance period.

The initial definition of a suspected case-patient from sentinel surveillance was any patient >5 years of age who reported no travel to a Zika-affected area within the previous 4 weeks and showed >3 of the 4 major Zika signs/symptoms: fever, rash, arthralgia, or conjunctivitis. Because children frequently have fever and rash, patients <5 years of age were excluded because of low specificity of the symptom-based case definition in this population (*16*). An exception was made for a 1-year-old patient who had all 4 signs/symptoms and reported no travel to a Zika-affected country.

### Enhanced Passive Surveillance

Enhanced passive surveillance is an amplification of standard passive surveillance in which public health agencies send notifications to healthcare providers and facilities to remain alert for suspected cases of a particular disease or condition (*19*). In July 2016, local transmission of Zika virus in Florida prompted the expansion of sentinel surveillance to enhanced passive surveillance starting in August 2016 (*20*). DOHMH used the Health Alert Network, an email-based public health alert system, to encourage all physicians to seek Zika virus testing for eligible patients. The final case definition for sentinel and passive sites was any patient who met all of the following criteria: >5 years of age; >3 of 4 signs/symptoms (arthralgia, fever, conjunctivitis, and rash); no history of travel to a Zika-affected area in the previous 4 weeks; no history of sex with a person who traveled to a Zika-affected area in the previous 4 weeks; and urine specimen collected within 14 days after illness onset.

### Routine Case Investigation

Following DOHMH protocol, epidemiologists interviewed patients who had laboratory evidence of Zika virus infection (or their guardians). During these interviews, investigators asked patients whether they had any nonsexual household contacts who developed Zika-like signs/symptoms and whether the contact had traveled to a Zika-affected area. Any reports of symptomatic, nonsexual household contacts without travel were assessed for risk and referred to testing if appropriate.

### Laboratory Testing

Patients who met the sentinel case definition were asked to provide >3 mL of urine in sterile tubes. According to CDC guidelines, urine must have been obtained within 14 days after illness onset to be eligible for testing (*21*). The urine samples were stored at 4°C until they could be transported on ice to the NYC DOHMH Public Health Laboratory (PHL) for testing. DOHMH arranged for transportation of specimens via a courier service. Pregnant patients were requested to submit not only urine specimens but also serum specimens. All sentinel specimens were prioritized (within 48 hours) for Zika virus RNA testing by a real-time reverse transcription PCR (rRT-PCR) assay at PHL (*22*). We assessed timeliness of PHL testing using the diagnosis time, defined as the number of hours between specimen collection at the healthcare facility and rRT-PCR result availability, as well as testing time, defined as the number of hours between specimen arrival at PHL and rRT-PCR result availability.

### Syndromic Surveillance

Syndromic surveillance uses electronic health-related data in near–real time to assess the health of a community with the goal of early identification of disease clusters or cases (*23*). The NYC DOHMH syndromic surveillance system uses daily visit data from all NYC EDs. For visits by patients >5 years of age, chief complaint text and International Classification of Diseases (ICD) version 9 and 10 diagnosis codes were used to create a fever syndrome for spatiotemporal analysis and a Zika-like illness line list for case finding.

Patients with fever visits were identified as those with chief complaint terms fever, febrile, or pyrexia or with ICD version 9 diagnosis code 780.6 or ICD version 10 diagnosis code R50. Fever was chosen as a surrogate marker for potential locally acquired Zika virus disease because fever was reported by ≈80% of symptomatic NYC patients with travel-associated Zika virus disease at the time of sign/symptom selection; is uncommon among persons >5 years of age during mosquito season in NYC; and was found to be more specific than rash, conjunctivitis, or arthralgia, with less variety in patient chief complaint.

Each day, we applied the prospective space-time permutation scan statistic using SaTScan version 9.4 invoked in batch mode through SAS version 9.4 to identify spatiotemporal clusters of fever syndrome by hospital ZIP code and by patient residential ZIP code (*24*). The input file was for 90 days, the maximum spatial cluster size was 20% of observed visits, and the maximum temporal cluster size was 14 days. Initially, we defined a signal as a cluster with a recurrence interval >100 days, but to limit false signaling, on June 18, 2016, we redefined a signal as a cluster with a recurrence interval >365 days. A recurrence interval represents the number of days of daily surveillance required for the expected number of clusters at least as unusual as the observed cluster to be equal to 1 by chance (*25*). We defined unique clusters as clusters with no spatial overlap with ZIP codes or hospitals identified in the prior day’s most likely cluster. We overlaid clusters on a map of areas identified using a statistical model as being at high risk of Zika virus importation in any given week. We evaluated spatiotemporal clusters qualitatively, taking into consideration the recurrence interval, whether hospitals included in the cluster recently transitioned to patient tracking and data transfer using Health Level Seven (HL7) international reporting standards (http://www.hl7.org), cluster size relative to the estimated <200 m range of the *Ae. albopictus* mosquito, and any geographic overlap with areas at high risk for Zika virus importation (*26*).

In addition, patients with Zika-like illness were identified through ED visits with mention of any the following scenarios: chief complaint including at least 3 signs/symptoms among rash, fever, joint pain, or conjunctivitis; chief complaint including the term Zika; diagnosis of Guillain-Barré syndrome; or diagnosis of arboviral infection. Initially, DOHMH staff members reviewed all ED visits for Zika-like illness. As the volume of visits increased through June and July, a case finding pilot was conducted to determine whether syndromic surveillance could identify nontravel patients for testing and, if so, establish rules for when follow-up investigation was necessary. During the pilot period, July 31–August 4, 2016, DOHMH surveillance analysts contacted hospital staff to collect information on travel history, diagnosis, and any Zika testing of patients identified as having Zika-like illness. Because this work was conducted in the course of routine surveillance and public health practice, institutional review board approval was not required.

## Results

### Sentinel and Enhanced Passive Surveillance

The NYC DOHMH sentinel surveillance system for locally acquired Zika virus infection was operational during June 27–September 30, 2016, and consisted of 24 NYC hospitals and community health centers. Sentinel sites were located in all 5 boroughs: 7 in the Bronx, 6 in Queens, 5 in Brooklyn, 3 in Manhattan, and 3 in Staten Island. Enhanced passive surveillance was instituted on August 2, 2016.

A total of 15 patients met the suspected case definition; of these, 8 (53%) were reported from 5 sentinel sites and 7 (47%) from 6 nonsentinel sites (Table), including 4 hospitals and 2 outpatient centers. The most common location of residence was the Bronx (n = 5), followed by Manhattan (n = 3) and Queens (n = 3). All patients from the Bronx sought care at sentinel sites. The median patient age was 35 years (interquartile range [IQR] 20–49 years). Nine (60%) patients were female. Two patients were pregnant at the time of testing. No household contacts of cases were referred for Zika testing during routine case investigation.

**Table:** Demographic and clinical characteristics of persons meeting case definition for suspicion of local transmission of Zika virus at sentinel and nonsentinel passive surveillance sites in New York City, June 27–September 30, 2016*

Characteristics	Total tested for Zika virus	Nonsentinel site	Sentinel site
Total number tested	15 (100)	7 (47)	8 (53)
Median age (interquartile range)	35 (20–49)	29 (9–38)	45 (21–54)
Borough			
Bronx	5 (33)	1 (14)	4 (50)
Brooklyn	2 (13)	1 (14)	1 (13)
Manhattan	3 (20)	2 (29)	1 (13)
Queens	3 (20)	3 (43)	0
Staten Island	2 (13)	0	2 (25)
Sex			
F	9 (60)	6 (86)	3 (38)
M	6 (40)	1 (14)	5 (62)
Pregnant at time of report	2 (13)	2 (29)	0
Signs and symptoms			
Arthralgia	12 (80)	4 (57)	8 (100)
Conjunctivitis	10 (67)	7 (100)	3 (38)
Fever	13 (86.7)	5 (71.4)	8 (100)
Rash	11 (73.3)	4 (57.1)	7 (87.5)

*Values are no. (%) except as indicated. Signs and symptoms are not mutually exclusive.

The median diagnosis time for all submissions was 46.3 hours (IQR 27–98 hours), and the median testing time was 6.2 hours (IQR 6–26 hours). No specimens had Zika virus RNA detected by rRT-PCR. 

### Fever Syndrome ED Visits

We performed automated spatiotemporal cluster detection analyses for fever syndrome daily on visits from all 53 NYC EDs during June 1–October 31, 2016. During this period, there were 40,073 visits for fever syndrome, with a daily median of 262 visits (IQR 247–277 visits). We detected 17 unique spatiotemporal fever syndrome clusters. Upon investigation, we discarded 2 clusters with low recurrence intervals, because these clusters would not have signaled after applying the final signaling threshold. We examined 14 other clusters that we identified as artifacts resulting from 17 hospitals transitioning during the analysis period to using HL7 international reporting standards. The transition resulted in longer fields for the chief complaint, and thus more opportunity to include a fever keyword, causing localized increased syndrome capture compared with the baseline. The remaining cluster did not overlap with areas classified as high risk for Zika importation and had a radius of 11.1 km, inconsistent with the estimated <200 m range of *Ae. albopictus* mosquitoes (*26*).

### Zika-Like Illness ED Visits

We identified 308 ED visits for Zika-like illness during June 1–October 31, 2016 (Figure); daily median was 2 visits (IQR 1–3 visits). During the case finding pilot, we identified 19 Zika-like illness visits at 17 hospitals. For 6 visits, DOHMH surveillance analysts could not reach hospital staff for follow-up after 3 attempts or medical records were unavailable. Of 13 (68%) visits with completed follow-up, travel to Zika-affected countries was confirmed for 85% of patients. The remaining 15% of patients with completed follow-up and no travel were found to have diagnoses inconsistent with Zika virus infection. Follow-up determined that all visits with mention of the term Zika were for patients with travel history and had been appropriately assessed for infection risk and testing by the ED physician.

**Figure:**
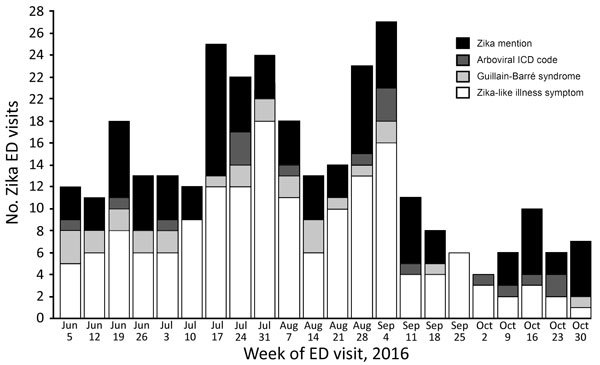
Number of ED visits for Zika-like illness in New York City, NY, USA, during June 1–October 31, 2016, by week and type of visit. ED, emergency department; ICD, International Classification of Diseases.

Based on the case finding pilot, we established that follow-up case investigation was not necessary when the visit text had one of the following: mention of recent travel to a country or territory with local Zika virus transmission, an alternate diagnosis listed, or a chief complaint containing “Zika.” Following this protocol, during August 5–October 31, 2016, a total of 12 of 163 Zika-like illness visits qualified for additional follow-up: 8 were found to have traveled to Zika-affected countries, but this information was not noted in the original chief complaint or travel history data fields; 1 patient had already been tested for Zika virus; 1 patient was ruled out because medical records noted allergic reaction with no fever; 1 patient was admitted to labor and delivery from the ED and the ED medical record was inaccessible to the hospital staff contact; and 1 patient could not be matched to hospital records on the basis of information available in the syndromic record.

## Discussion

During the peak period of travel-associated Zika cases and the mosquito season in NYC in 2016, DOHMH used case investigation, sentinel, enhanced passive, and syndromic surveillance to monitor for the presence of local mosquitoborne Zika virus transmission. None of the systems detected any local mosquitoborne transmission.

During the sentinel surveillance period, healthcare providers identified 15 NYC residents without recent travel history or sexual exposure who met the sentinel case definition for Zika virus; all tested negative for Zika virus. Only 21% of sentinel sites reported any suspected cases, which might indicate a lack of engagement of some sites, inappropriate selection of site locations, or a true lack of patients who met the criteria. Future iterations of a sentinel surveillance program should include an evaluation component, such as the deployment of a mock patient with locally acquired Zika virus infection or a review of medical records, to evaluate provider awareness and effectiveness of the sentinel sites.

Although DOHMH targeted efforts to identify suspected locally acquired cases at presumed higher-risk sentinel sites, 47% of patients tested for Zika virus were seen at nonsentinel sites. We believe this might have been because Zika virus infection is a reportable disease in New York and healthcare facilities were already sending specimens from suspected travel-associated Zika virus cases to PHL for testing (*27*). Given that baseline knowledge regarding Zika may have been heightened, the additional contact with sentinel sites may not have appreciably improved reporting.

Enhanced passive citywide surveillance has the advantages of including all NYC residents and relying on the preexisting reportable disease system. Potential downsides to this open system are the incorrect application of screening criteria and subsequent overburdening of laboratories with test requests. In response to the high volume of travel-associated Zika virus testing through PHL, by March 2016 DOHMH had implemented a system to screen testing requests for appropriateness (*15*). This same system was used to screen test requests of suspected local transmission, mitigating the potential for incorrect application of screening criteria.

CDC guidelines for early detection of possible local transmission require timely testing of patients (*16*). For both sentinel and passive surveillance, the use of urine specimens for Zika virus testing was less invasive for patients and allowed health centers without laboratory capabilities to submit specimens, because serum testing requires specimen processing with a centrifuge shortly after collection. DOHMH also arranged for the transport of specimens for testing to PHL, reducing provider burden. The median time between specimen collection and result availability was 46 hours, indicating that PHL was able to efficiently process and report suspected cases of local mosquitoborne transmission. The caveat to testing urine only is that Zika virus RNA may persist longer in serum than in urine (*28*).

During June–October 2016, no concerning spatiotemporal clusters of fever were identified through syndromic surveillance. Because available spatial elements of the data were limited to hospital location and patient residential ZIP code, spatiotemporal clusters associated with patient workplace were not detectable. In addition, although the initial choice of fever syndrome alone was appropriate for NYC at the start of the case finding, by the end of the study period, rash was identified as the most prevalent sign or symptom of Zika virus infection. This finding is in line with a CDC study of travel-associated cases during January–March 2016 that found that rash was the most common sign or symptom reported (98%), followed by fever (82%) and arthralgia (66%) (*29*). Requiring >2 signs/symptoms, rather than >3, would have been less sensitive for cluster detection because of patients who experience or report only 1 relevant sign/symptom and given the restrictions in the length of the chief complaint text provided by some hospitals. Although the incidence of other febrile illnesses during the summer in NYC is low, particularly compared with illnesses with rash, evaluation on the basis of fever syndrome may not be as appropriate in tropical settings where high incidence of febrile illnesses might circulate concurrently with Zika virus. Rash should be evaluated for future syndromic surveillance, with consideration for potential background levels of rash during the summer.

During August–October 2016, a manageable number (12 of 163) of ED visits for Zika-like illness met protocol criteria for DOHMH staff to follow up with hospital infection control practitioners regarding patient travel history. Follow-up activities would be challenging to sustain with a higher volume of cases. The pilot study highlighted the importance of collecting travel history data and led DOHMH to request during the study period that clinical facilities include an additional travel history field in the daily ED data transmission to improve capture of patient travel. Continued collaboration between DOHMH and EDs to improve travel data will facilitate surveillance for Zika virus as well as for other travel-associated diseases.

A limitation of the syndromic surveillance system was the incomplete transition of all hospitals to HL7 international reporting standards, resulting in differences in the average number of terms in the ED chief complaint per hospital and precluding use of a more specific, multiple-symptom definition for cluster detection. For the Zika-like illness linelist, requiring at least 3 signs/symptoms of Zika virus infection biased case detection toward hospitals that provide more detailed chief complaints, which might not have corresponded to areas where the risk of local mosquitoborne transmission was highest in NYC.

The major limitation of all surveillance for local transmission of Zika virus is that 80% of Zika virus infections are asymptomatic; therefore, any surveillance system reliant on the presence of symptoms will underestimate the true incidence of infection (*4**,**30*). Thus, the surveillance systems used by DOHMH might have missed capturing smaller, local outbreaks of mosquitoborne Zika virus. In particular, spatiotemporal cluster detection requires many symptomatic persons seeking care within a specified interval. Therefore, it is unlikely that syndromic surveillance would be sufficiently sensitive to detect a small cluster of locally acquired infections.

Despite the stated limitations, our experience suggests that enhanced passive surveillance, with frequent outreach to providers in communities with large numbers of travel-associated human cases or habitats conducive to potentially competent mosquito vectors, was an efficient and manageable method to monitor for locally transmitted mosquitoborne Zika virus infection in NYC. Given that NYC does not have *Ae. aegypti* mosquitoes, the multipronged surveillance approach taken by NYC DOHMH during the first year of the epidemic was considered a robust plan to detect local mosquito transmission of Zika virus. The likelihood that another vectorborne infection will emerge somewhere in the world is high, and surveillance is a critical tool for the detection and evaluation of control measures (*31**,**32*). Our experience offers a possible surveillance model for other jurisdictions concerned about the possibility of local mosquitoborne Zika virus or other arboviral transmission.
